# Overcoming the age-dependent SARS-CoV-2 vaccine response through hybrid immunity: analysis of humoral and cellular immunity with mass cytometry profiling

**DOI:** 10.1186/s12979-024-00454-z

**Published:** 2024-07-30

**Authors:** Zayakhuu Gerelkhuu, Sehee Park, Kyoung Hwa Lee, Yong Chan Kim, Sook Jin Kwon, Kyoung-Ho Song, Eu Suk Kim, Young Goo Song, Yoon Soo Park, Jin Young Ahn, Jun Yong Choi, Won Suk Choi, Seongman Bae, Sung-Han Kim, Shin-Woo Kim, Ki Tae Kwon, Hye Won Jeong, Kyong Ran Peck, Eun-Suk Kang, June-Young Koh, Jae-Hoon Ko, Tae Hyun Yoon

**Affiliations:** 1 Department of Chemistry, Research Institute for Convergence of Basic Science, Hanyang University, Seoul, Republic of Korea; 2 Department of Chemistry, College of Natural Sciences, Hanyang University, Seoul, Republic of Korea; 3 Division of Infectious Diseases, Department of Internal Medicine, Gangnam Severance Hospital, Yonsei University College of Medicine, Seoul, Republic of Korea; 4 Division of Infectious Diseases, Department of Internal Medicine, Yongin Severance Hospital, Yonsei University College of Medicine, Yongin, Republic of Korea; 5 Yoon Idea Lab. Co. Ltd, Seoul, Republic of Korea; 6 Department of Internal Medicine, Seoul National University Bundang Hospital, Seoul National University College of Medicine, Seongnam, Republic of Korea; 7 Department of Internal Medicine, Severance Hospital, Yonsei University College of Medicine, Seoul, Republic of Korea; 8 Division of Infectious Diseases, Department of Internal Medicine, Korea University Ansan Hospital, Korea University College of Medicine, Ansan, Republic of Korea; 9 Department of Infectious Diseases, Asan Medical Center, University of Ulsan College of Medicine, Seoul, Republic of Korea; 10 Department of Internal Medicine, School of Medicine, Kyungpook National University, Daegu, Republic of Korea; 11 Division of Infectious Diseases, Department of Internal Medicine, Kyungpook National University Chilgok Hospital, School of Medicine, Kyungpook National University, Daegu, Republic of Korea; 12 Department of Internal Medicine, Chungbuk National University College of Medicine, Cheongju, Republic of Korea; 13 Division of Infectious Diseases, Department of Medicine, Samsung Medical Center, Sungkyunkwan University School of Medicine, Seoul, 06351 Republic of Korea; 14 Department of Laboratory Medicine and Genetics, Samsung Medical Center, Sungkyunkwan University School of Medicine, Seoul, Republic of Korea; 15 Genome Insight, Inc, La Jolla, San Diego, CA USA; 16 Department of Medical and Digital Engineering, College of Engineering, Hanyang University, Seoul, Republic of Korea; 17 Institute of Next Generation Material Design, Hanyang University, Seoul, Republic of Korea

**Keywords:** Aging, COVID-19 vaccines, Mass cytometry, Immunity

## Abstract

**Background:**

Age-dependent immune responses to coronavirus disease 2019 (COVID-19) vaccinations and breakthrough infections (BIs) in young and middle-aged individuals are unclear.

**Methods:**

This nationwide multicenter prospective cohort study analyzed immune responses in participants of the ChAdOx1 (ChAd)-ChAd-mRNA vaccine group using cytometry by time-of-flight, anti-spike protein antibody (Sab) and anti-nucleocapsid antibody (Nab) titers, plaque reduction neutralization tests (PRNTs), and interferon-gamma (IFN-γ) release assays at various time points.

**Results:**

We evaluated 347 participants with an average age of 38.9 ± 9.4 years (range: 21–63). There was a significant inverse correlation between age and Sab levels after the second dose (slope − 14.96, *P* = 0.032), and this was more pronounced after the third dose (slope − 208.9, *P* < 0.001). After BIs, older participants showed significantly higher Sab titers (slope 398.8, *P* = 0.001), reversing the age-related decline observed post-vaccination. This reversal was also observed in PRNTs against wild-type SARS-CoV-2 and the BA.1 and BA.5 variants. IFN-γ responses increased markedly after the third dose and Bis, but showed a weak positive correlation with age, without statistical significance. Immune cell profiling revealed an age-dependent decrease in the proportions of B-cell lineage cells. The proportions of naive CD4^+^ and CD8^+^ T cells were inversely correlated with age, whereas the proportions of mature T cell subsets with memory function, including memory CD4^+^ T, CD8^+^ T_EM_, CD8^+^ T_EMRA_, and T_FH_ cells, increased with age.

**Conclusions:**

Age-dependent waning of the serologic response to COVID-19 vaccines occurred even in middle-aged individuals, but was reversed after BIs. IFN-γ responses were preserved, compensating for the decrease in naive T cell populations, with an increase in memory T cell populations.

**Supplementary Information:**

The online version contains supplementary material available at 10.1186/s12979-024-00454-z.

## Background

Vaccination plays a crucial role in managing infectious diseases, and understanding the immune response to each vaccine platform is crucial for establishing a vaccination strategy. In particular, research on vaccine effectiveness across different age groups is essential for personalized vaccination. While much attention has been given to the low immunogenicity among older populations [1], there is a limited understanding of immune response variability in middle-aged individuals compared with young individuals [2–4]. The coronavirus disease 2019 (COVID-19) pandemic, caused by severe acute respiratory syndrome coronavirus 2 (SARS-CoV-2), has prompted an unprecedented global effort to develop and distribute vaccines. The main COVID-19 vaccination is advised for adults of all ages at the same time, in contrast to other vaccines that are suggested for particular age groups based on the risk. This effort has provided an opportunity to study the serial immune response following vaccination and breakthrough infections (BIs) in subjects without pre-existing immunity.

Previous studies have shown that older adults tend to exhibit a weaker humoral and cellular immune response to vaccination, which can lead to reduced vaccine efficacy. For instance, Wang et al [1]. highlighted the impact of age on the efficacy and safety of COVID-19 vaccines, emphasizing the need for tailored vaccination strategies for older populations. However, the immunogenicity in middle-aged individuals remains less understood, leaving a gap in our knowledge of how vaccine responses change from young adulthood into middle age [2]. Understanding these dynamics is crucial as it can inform recommendations for booster shots and optimize vaccine formulations to cater to different age groups [4].

This study aimed to evaluate age-dependent immune responses following COVID-19 vaccination and BI. This has become particularly pertinent as the COVID-19 pandemic enters the global endemic era, characterized by vaccination and BI in most populations. These data provide crucial evidence for the necessity of annual booster shot administration in middle-aged individuals, which remains controversial [5]. We conducted immune cell profiling using cytometry by time-of-flight (CyTOF) technique, along with assessments of anti-spike protein antibody (Sab) and anti-nucleocapsid antibody (Nab) titers and the results of plaque reduction neutralization tests (PRNTs) and interferon-gamma releasing assays (IGRAs), to estimate the overall landscape of immune responses. The study was conducted in a homogeneous cohort of healthy healthcare workers (HCWs) without a history of SARS-CoV-2 infection, who received the ChAdOx1 (ChAd; AstraZeneca, Cambridge, UK) primary vaccine series, followed by mRNA booster vaccinations. Subsequently, we analyzed BIs through a longitudinal follow-up study.

Our comprehensive approach, which includes CyTOF profiling, antibody titers, and functional assays, provides a detailed analysis of the immune response across different age groups. By focusing on middle-aged individuals, our study fills a critical gap in understanding how vaccine-induced immunity wanes and recovers with age. This information is particularly valuable for informing public health strategies and vaccination policies as the world adapts to the endemic presence of COVID-19. Additionally, our findings underscore the importance of personalized vaccination strategies to enhance immune protection in middle-aged populations, especially those with underlying health conditions that may further compromise their immune response.

## Methods

### Study population and specimen collection

This study was part of a nationwide multicenter prospective cohort study conducted in the Republic of Korea to assess the immunogenicity of COVID-19 vaccines under research funding from the Korea Disease Control and Prevention Agency (KCDA). Cohort data were analyzed and published at various points during the pandemic to meet on-demand needs [6–13]. In the Republic of Korea, the primary COVID-19 vaccine series included ChAd or mRNA vaccines (either BNT162b2; Pfizer, New York, NY, USA or mRNA-1273; Moderna, Cambridge, MA, USA), with booster shots consisting of mRNA vaccines. To analyze the effect of age on vaccine-induced immunity, we controlled for the type of vaccine administered, focusing on the ChAd-ChAd-mRNA group, which had a high proportion of middle-aged individuals. Individuals with a prior infection before mRNA booster administration, confirmed by polymerase chain reaction (PCR), or positivity for Nab, were excluded [14]. Sab was universally measured at all sampling points, whereas IGRA, PRNT, and CyTOF immune profiling focused on the time points before and after the third dose. This study was approved by the Institutional Review Board (IRB) of each institution and written informed consent was obtained from all participants.

### Laboratory procedures

Detailed procedures for assessing immunogenicity, including measurements of Sab, Nab, IGRA, and PRNT, have been described in previous studies [15–19]. In brief, Elecsys anti-SARS-CoV-2 S kits (Roche Diagnostics, Basel, Switzerland) were used for the quantitative measurement of Sab, with dilutions applied up to a 1:200 ratio, and the test results were converted to binding antibody units (BAU) per mL [15, 20, 21]. For Nab measurements, an Elecsys anti-SARS-CoV-2 kit (Roche Diagnostics) was utilized. Its performance in detecting natural infections has been validated in prior studies [15, 17]. The cellular immune response was assessed using a SARS-CoV-2–specific IGRA kit with an enzyme-linked immunosorbent assay (ELISA) (Covi-FERON ELISA; SD Biosensor, Suwon, Republic of Korea) [19]. This kit, which was developed during the early stages of the pandemic, utilizes spike proteins derived from wild-type (WT) SARS-CoV-2 and alpha variants for stimulating antigens. PRNTs were conducted for the WT, BA.1, and BA.5 strains of SARS-CoV‐2 at KDCA. The 50% neutralizing dose (ND_50_) was calculated using the Karber formula [22]. Hepatitis B surface antigen (HBsAg) and antibody (HBsAb) titers were measured using Elecsys HBsAg II and anti-HBs II kits, respectively (Roche Diagnostics).

### CyTOF analysis

For immune cell profiling, peripheral blood mononuclear cells (PBMCs) from a subset of HCWs were analyzed using high-dimensional mass cytometry at the single-cell level. Based on the Sab levels and IGRA results, 30 donors were selected to represent good and poor responders and analyzed with CyTOF. Briefly [23–25], whole blood from 30 donors was taken in EDTA-treated tubes at two different time points and PBMCs were isolated from whole blood using the density gradient method by Ficoll. The cells were first stained with a panel of 21 surface markers and 5 intracellular markers (Table S1 for the complete marker list). Additionally, a recombinant SARS-CoV-2 receptor-binding domain (RBD) tetramer labeled with 172Yb was used to identify spike-specific B cells. According to the manufacturer’s instructions, after staining, cells were fixed with 4% paraformaldehyde (PFA) and stained with an iridium-containing DNA intercalator. The cells were washed with cell acquisition solution (CAS) and analyzed using a Helios mass cytometer (Standard BioTools, South San Francisco, CA, USA) in CAS supplemented with 1× EQ four-element calibration beads. We analyzed donors’ phenotypic differences before and after booster shot vaccinations by manually gating 22 immune cell types (Additional file 1).

### Statistical analysis

Linear regression was utilized for correlation analyses between two continuous variables, particularly focusing on age and other variables of interest. We used linear regression to calculate slopes and visualize the data. Additionally, due to the possibility that some variables did not follow a normal distribution, Spearman’s correlation was also applied and presented. Given that multiple tests were conducted to identify age-related correlations with various immune markers, Bonferroni correction was applied to Spearman’s correlation, with α = 0.05/n, where n represents the number of tests in different analysis sets. The chi-square test was used to compare the IGRA responses using a quadrant chart. All *P* values were two-tailed, and a value of *P* < 0.05 was considered statistically significant. GraphPad Prism version 10.2.0 (GraphPad Software, San Diego, CA, USA) was used to create graphical representations and conduct the statistical analyses. For CyTOF profiling, flow cytometry standard files were bead-normalized prior to data export. Data were processed using FlowJo software (version 10.8.0; FlowJo, LLC, Ashland, OR, USA). Raw data were transformed into inverse hyperbolic sines, and manual gating (Additional file 1) was employed to identify cell types based on surface markers. Visualization of high-dimensional mass cytometry data at a single-cell resolution was achieved using a uniform manifold approximation and projection technique.

## Results

### Baseline characteristics of the study population

In total, 347 HCWs were included in the analysis as summarized in Table 1. The study population had a higher proportion of females (76.4%) than males (23.6%), with an average age of 38.9 ± 9.4 years. Their ages ranged from 21 to 63 years. Older individuals could not be included because of retirement. Among them, 73 (21.0%) were in their 20s, 114 (32.9%) were in their 30s, 103 (29.7%) were in their 40s, and 57 (16.4%) were in their 50s and 60s. The participants were generally healthy. The average body mass index (BMI) was 22.3 ± 2.8 kg/m^2^, with only 2.0% having a BMI greater than 30 kg/m^2^. Underlying diseases were reported in 11.8% of the participants, with the most common being hypertension (2.9%), followed by uncomplicated diabetes mellitus (1.7%) and hypothyroidism (1.7%). A more detailed distribution of samples across different age groups is provided in Table S2. All participants received the first dose of the ChAd vaccine in March 2021 and the second dose from May to June 2021, with a 12-week interval from the first dose. The third doses of the mRNA vaccine were administered from November 2021 to January 2022 according to the policies of the participating centers. A total of 2,414 specimens from the following seven sampling points were analyzed to investigate age-dependent immune responses: (1) 3 weeks after the first dose (*n* = 347), (2) 2 weeks after the second dose (*n* = 347), (3) 6 months after the second dose (*n* = 341; before the third dose), (4) 1 month after the third dose (*n* = 335), (5) 3 months after the third dose (*n* = 343; including 37 cases of BI, 10.8%), (6) 6 months after the third dose (*n* = 335; including 186 cases of BI, 55.5%), and (7) 9 months after the third dose (*n* = 366; including 263 cases of BI, 71.9%). Because all participants were healthy and vaccinated at the time of the BI, individuals who experienced BI fully recovered from mild illness, and none progressed to severe disease or required hospitalization. There were nine dropouts during the study period and some were missing sampling points due to personal situations.

**Table 1:** Participant demographics

Characteristics	Description/Value
Total participants	*n* = 347
Gender	Female: 265 (76.4%) Male: 82 (23.6%)
Age, years (average ± SD)	38.9 ± 9.4
BMI (average ± SD) kg/m^2^	22.3 ± 2.84 2.0% with BMI > 30
Underlying diseases %	11.8% - Hypertension: 2.9% - Diabetes mellitus: 1.7% - Hypothyroidism: 1.7%
Vaccine series	ChAdOx1 (AstraZeneca) primary series, mRNA booster
Time points for sampling	1. 3 weeks after 1st dose 2. 2 weeks after 2nd dose 3. 6 months after 2nd dose (before 3rd dose) 4. 1 month after 3rd dose (after 3rd dose) 5. 3 months after 3rd dose 6. 6 months after 3rd dose 7. 9 months after 3rd dose
Breakthrough Infections (BIs)	10.8% at 3 months post-3rd dose 55.5% at 6 months post-3rd dose 71.9% at 9 months post-3rd dose
Health Status Post-BI	Mild illness, full recovery, no severe cases or hospitalizations

### Age-dependent immunogenicity to vaccination and BI

**Fig. 1:**
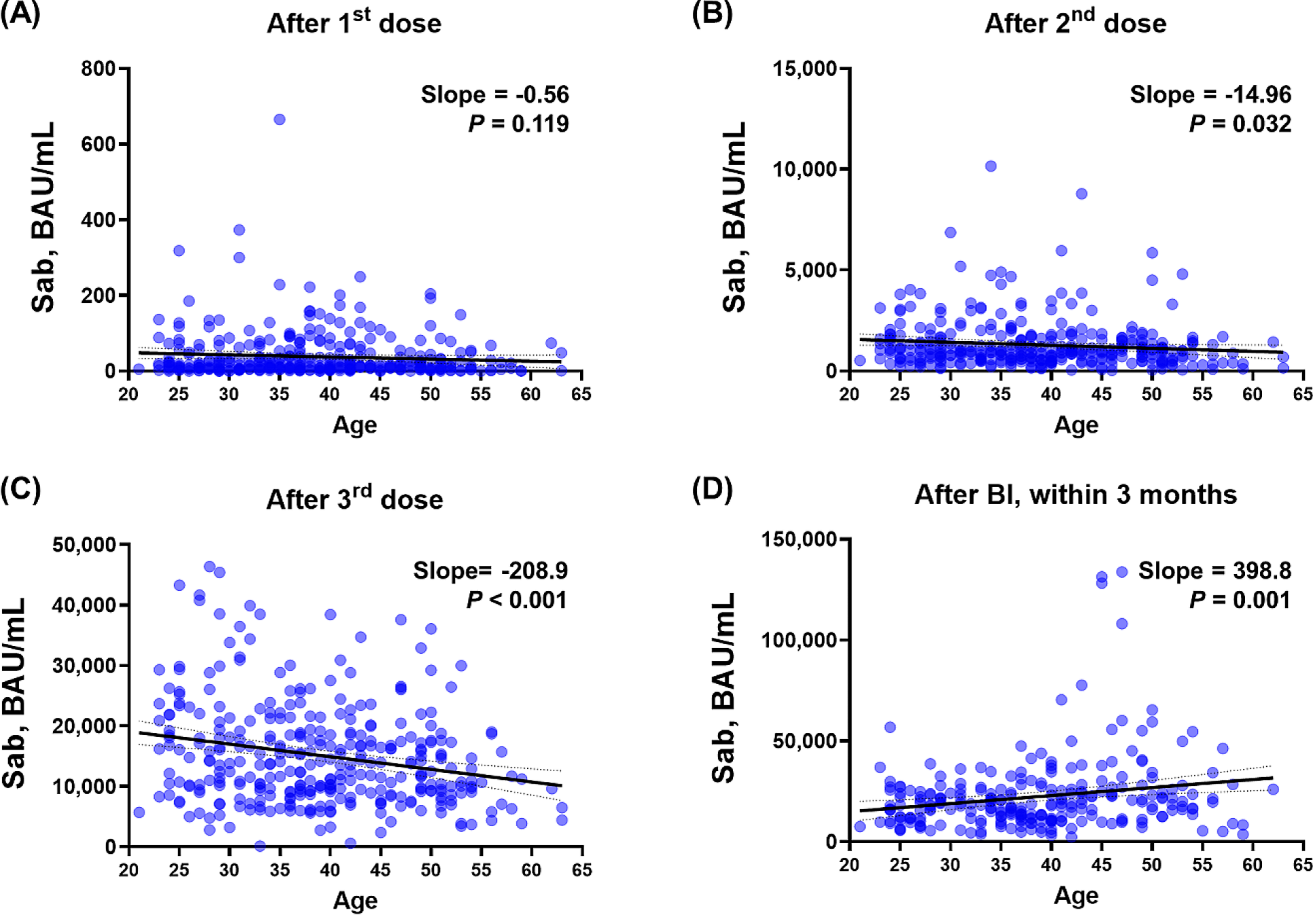
Age-dependent Sab response after COVID-19 vaccinations and BI. Correlation analysis between age and Sab titers measured at various time points. (**A**) after the first dose, (**B**) after the second dose, (**C**) after the third dose, and (**D**) after BIs. Each graph shows the slope of the correlation and the *P* value, indicating the strength and significance of the relationship between age and Sab levels. Abbreviations Sab, anti-spike-protein antibody; BAU, binding antibody unit; COVID-19, coronavirus disease 2019; BI, breakthrough infection

Quantile-quantile (Q-Q) plots were generated for Sab post-vaccination, as Additional file 2 illustrates. Despite the Q-Q plot’s overall linear appearance, there were a few variations from the predicted fitness. Consequently, in addition to linear regression, we applied Spearman’s correlation to all main figure sets, summarized the results, and presented them in Table S3. Overall, the significance of the *P* values from both linear regression and Spearman’s correlation was similar, allowing us to provide more precise statistical measures. As stated above, while the overall trend appeared linear, some deviations were observed. Therefore, we conducted a Spearman correlation analysis, and the significance of the *P* values was observed to be similar. To investigate age-dependent immune responses to COVID-19 vaccinations and subsequent BIs, we initially analyzed the correlation between age and Sab levels after each antigen-stimulating event (Fig. 1 and Additional file 3 A). After the first dose of vaccination, an inverse linear correlation was observed (slope − 0.56) without statistical significance (*P* = 0.119). The inverse linear correlation became significant (slope − 14.96, *P* = 0.032) after the second dose of vaccination and more pronounced after the third dose (slope − 208.9, *P* < 0.001). However, following BIs, older HCWs exhibited significantly higher Sab titers with a positive linear correlation (slope 398.8, *P* = 0.001), reversing the age-related decline in immune responses after vaccination. In addition, the Bonferroni correction method was used to correct the relationships of the multiple comparisons (Table S3). After this correction, Fig. 1B is no longer significant, while Fig. 1C and D remain significant. At the waning points, the inverse linear correlation after the third dose of vaccination and the positive linear correlation after BI remained, albeit with decreasing absolute slope values (Additional file 4). Although statistical significance was not achieved in the correlation analysis between age and PRNT titers, owing to the limited number of tested participants (*n* = 32), an inverse linear correlation was observed in the WT PRNT after the third dose of the vaccine. However, age-dependent correlations were not observed for the BA.1 PRNT or BA.5 PRNT titers, but a positive correlation after BI was observed for all PRNTs ( Additional file 5). Nab levels did not exhibit a clear age-dependent correlation after BI (Additional file 6).

To determine the potential confounding factors, we further investigated other clinical factors that may affect Sab titers in addition to age. First, the association between responses to previous antigenic stimulations and subsequent stimulations was examined using log_10_ values (Additional file 7). Sab titers measured after the first dose of vaccination exhibited a significant positive linear correlation with those measured after the second dose (slope = 0.25, *P* < 0.001). Both Sab titers measured after the third dose and BI exhibited statistically significant positive linear correlations, but the correlation gradually diminished, as indicated by decreasing slopes (0.18 and 0.08, respectively). Similarly, Sab titers measured after the second dose exhibited a stronger correlation with those measured after the third dose and weakened after BI (slope = 0.45 and 0.16, respectively). Second, to investigate potential associations with immunogenicity induced by other vaccines, we measured HBsAb titers, as the hepatitis B vaccine is administered during childhood as part of the National Immunization Program. The analysis was conducted after excluding HCWs with positive HBsAg results, and no noticeable correlation was found between HbsAb titers and Sab titers or age (Additional file 8). Finally, sex-based differences were explored, as females tended to be younger in the HCW cohort (Additional file 9). Females exhibited significantly higher Sab titers after the second and third doses of vaccination. However, the difference was diminished after age matching (up to 1:2 matching within 1 year’s difference; male, *n* = 49, age = 39.7 ± 8.3; female, *n* = 93, age = 39.4 ± 7.9) and no significant difference between sexes was observed.

**Fig. 2:**
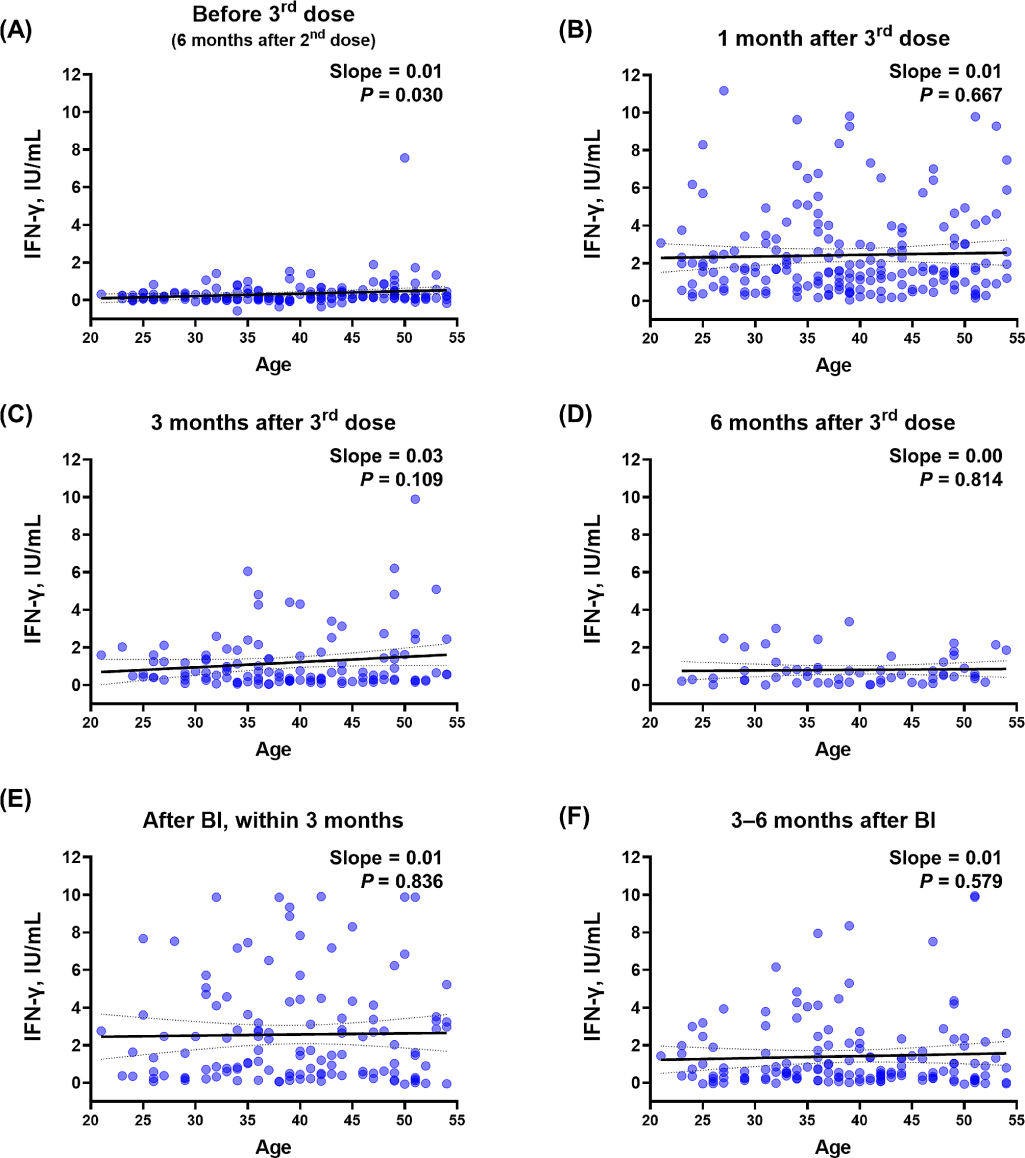
Age-dependent IGRA response after COVID-19 vaccinations and BI. Correlation between age and IFN-γ responses measured using an IGRA at various time points. (**A**) before the third dose, (**B**) 1 month after the third dose, (**C**) 3 months after the third dose, (**D**) 6 months after the third dose, (**E**) after BIs, and (**F**) 3–6 months after BIs. Each graph shows the slope of the correlation and the *P* value. Abbreviations IFN, interferon; IU, international unit; IGRA, interferon-gamma releasing assay; COVID-19, coronavirus disease 2019; BI, breakthrough infection

Next, to evaluate the age-dependent cellular immune response, we analyzed the interferon-gamma (IFN-γ) response to spike protein stimulation utilizing an IGRA kit (Fig. 2 and Additional file 3B). IFN-γ responses markedly increased after the third dose of vaccination and BI, but age-dependent correlations were not clearly observed overall. Although all the slopes indicated a weak positive correlation and statistical significance was noticed at the resting status before the third dose (*P* = 0.030, Fig. 2A), the trend was not pronounced after antigenic stimulating events (Fig. 2B and E). When the proportion of good and poor responders divided by the median values was compared at each antigenic stimulation event, there were no statistically significant differences between the young and old age groups (Additional file 10). Additionally, the relationships between the multiple comparisons are corrected using the Bonferroni-corrected *P* value summarized in Table S3. method. But they demonstrate that not significant.

### Immune cell profiling according to age, using multi-parametric mass cytometry analysis

**Fig. 3:**
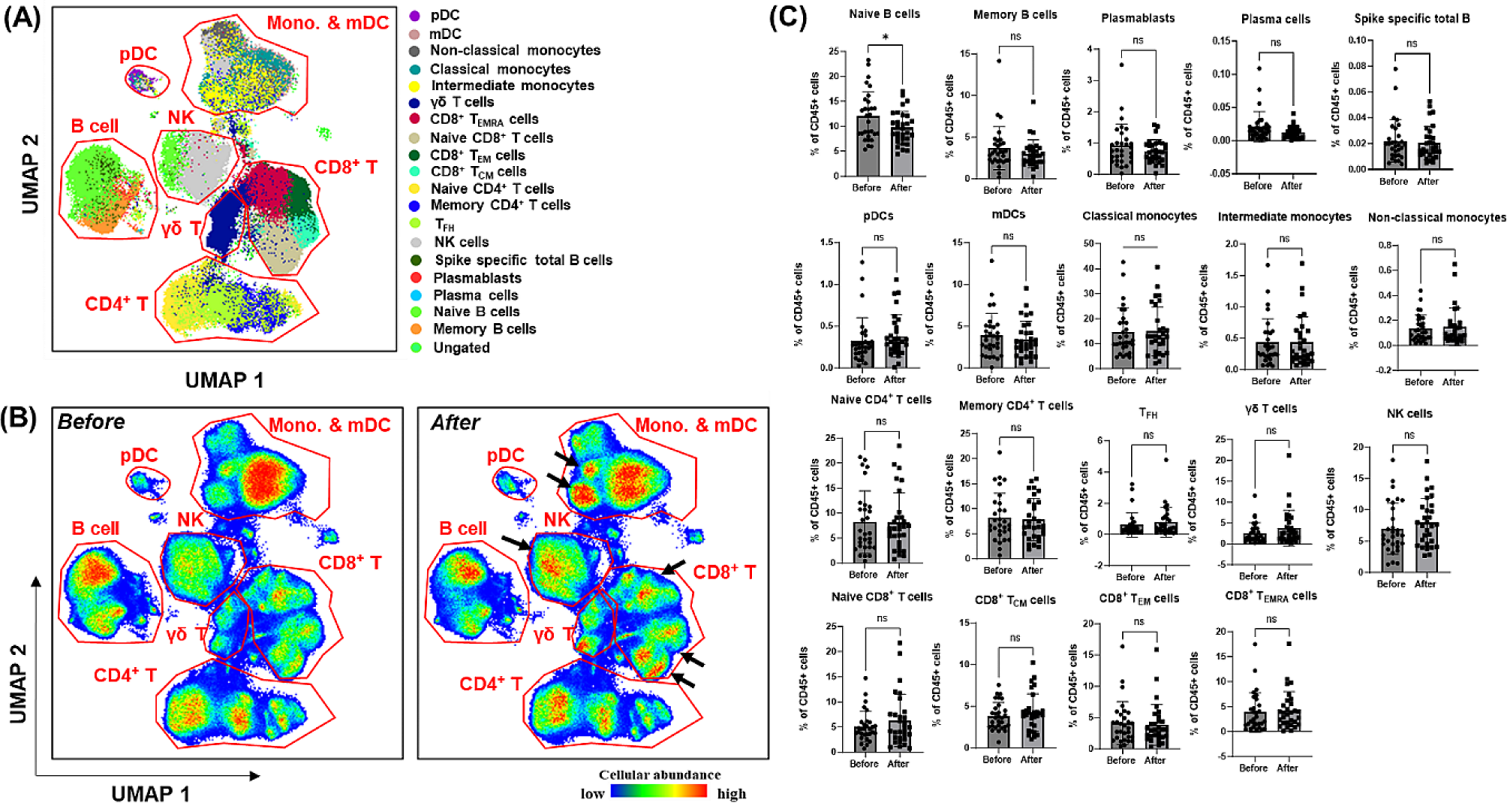
Immune cell profiles before and after the third dose of vaccination (**A**) UMAP visualization of total immune cells, (**B**) UMAP visualization of immune cells sampled before and after the third dose of vaccination, highlighting the differences in immune cell populations. (**C**) statistical comparison of each cell type between the two sampling points, including proportions, and significant changes. Abbreviations UMAP, uniform manifold approximation and projection; pDC, plasmacytoid dendritic cell; mDC, myeloid dendritic cell; CD, clusters of differentiation; NK, natural killer; T_FH_, follicular helper T; CM, central memory; EM, effector memory; TEMRA, terminally differentiated effector memory T cell re-expressing CD45RA

To uncover the underlying immune landscape that may elucidate age-dependent immune responses before and after the third dose of COVID-19 vaccinations and BI, we conducted immune cell profiling using multiparametric CyTOF analysis. Table 2 and Additional file 11 represent immune cell profiling at two specific time points: (1) immediately before the third dose (booster) and (2) one month after the third dose and their responses at these critical points in the vaccination schedule, providing insights into how the immune system adapts to booster vaccination. The detailed profiling helps to identify changes in various immune cell subsets, including B cells, T cells, and other major immune cell types. Additionally, we conducted Spearman’s correlation, which showed no significant difference in terms of statistical significance. The proportion of myeloid cells, including classical/intermediate/non-classical monocytes, and myeloid/plasmacytoid dendritic cells, natural killer cells, and γδ T cells, did not exhibit a clear correlation with age. The proportion of B cell lineages, including naive B cells, memory B cells, plasma cells, plasmablasts, and spike-specific B cells, displayed a weak declining trend with age, supporting the decreased antibody response observed in the middle-aged group. A contrasting phenomenon was observed for T cell lineages. The proportion of naive T cells showed a clear inverse correlation with age. In particular, the age-dependent decline in the proportion of naive CD8^+^ T cells (*P* = 0.013) was significant. Meanwhile, the proportion of mature T cell subsets with memory functions, including memory CD4^+^ T cells, CD8^+^ central memory T (T_CM_) cells, CD8^+^ effector memory T (T_EM_) cells, CD8^+^ terminally differentiated effector memory T cells re-expressing CD45RA (T_EMRA_), and follicular helper T (T_FH_) cells, demonstrated an increasing trend with age. Additionally, UMAP visualizations (Fig. 3A, B) and quantitative analysis (Fig. 3C) of immune cell profiles before and after the third dose were conducted for age-dependent groups, including middle-aged and young individuals. We also illustrated Sab levels in groups with low and high Sab levels in Additional file 12. Differences in immune cell profiles between these age groups showed distinct patterns before the third dose, but no significant differences were observed after. Spearman correlation with Bonferroni-corrected *P* values was calculated and showed non-significance, as detailed in Table S3.

**Table 2:** Correlation between age and immune cell subtypes collected before the third dose

Correlating variables	Linear regression		Spearman correlation		
	Slope	*P* value	Spearman’s ρ	*P* value	Bonferroni-corrected *P* value
Age-Classical monocytes	0.058	0.681	0.047	0.806	1.000
Age-Non-classical monocytes	0.003	0.898	-0.113	0.552	1.000
Age-mDCs	-0.002	0.957	-0.052	0.784	1.000
Age-pDCs	-0.004	0.367	0.035	0.856	1.000
Age-NK cells	-0.007	0.941	-0.013	0.948	1.000
Age-Naive B cells	-0.050	0.642	-0.209	0.268	1.000
Age-Memory B cells	-0.020	0.717	-0.144	0.447	1.000
Age-Plasma cells	-0.001	0.095	-0.314	0.091	1.000
Age-Naive CD4^+^ T cells	-0.117	0.385	-0.115	0.544	1.000
Age-Memory CD4^+^ T cells	0.150	0.144	0.201	0.287	1.000
Age-T_FH_ cells	0.028	0.095	0.089	0.640	1.000
Age-γδ cells	-0.001	0.979	-0.161	0.397	1.000
Age-Naive CD8^+^ T cells	0.155	0.013	-0.407	0.026	0.494
Age-CD8^+^ T_CM_ cells	0.009	0.795	0.037	0.848	1.000
Age-CD8^+^T_EM_ cells	0.089	0.222	0.323	0.082	1.000
Age-CD8^+^ T_EMRA_ cells	0.136	0.099	0.412	0.024	0.456
Age-Intermediate monocytes	0.063	0.228	0.084	0.523	1.000
Age-Spike-specific B cells	0.000	0.238	-0.302	0.019	0.361
Age-Plasmablasts	-0.016	0.057	-0.232	0.074	1.000

Abbreviations mDC, myeloid dendritic cell; pDC, plasmacytoid dendritic cell; NK, natural killer; CD, clusters of differentiation; T_FH_, follicular helper T; CM, central memory; EM, effector memory; T_EMRA_, terminally differentiated effector memory T cell re-expressing CD45RA

**Table 3:** Correlation between Sab and immune cell subtypes collected before the third dose

Correlating variables	Linear regression		Spearman correlation		
	Slope	*P* value	Spearman’s ρ	*P* value	Bonferroni-corrected *P* value
Sab-Classical monocytes	184.9	0.542	0.188	0.321	1.000
Sab-Non-classical monocytes	2138	0.183	0.299	0.109	1.000
Sab-mDCs	970.5	0.322	0.221	0.240	1.000
Sab-pDCs	-4768	0.582	-0.059	0.759	1.000
Sab-NK cells	88.74	0.853	0.004	0.984	1.000
Sab-Naive B cells	41.93	0.916	0.000	0.999	1.000
Sab-Memory B cells	242.2	0.751	0.058	0.762	1.000
Sab-Plasma cells	-30,302	0.725	0.075	0.694	1.000
Sab-Naive CD4^+^ T cells	-629.5	0.038	-0.286	0.126	1.000
Sab-Memory CD4^+^ T cells	-545.5	0.177	-0.259	0.167	1.000
Sab-T_FH_ cells	-4447	0.071	-0.220	0.244	1.000
Sab-γδ cells	-650.2	0.416	-0.064	0.736	1.000
Sab-Naive CD8^+^ T cells	-91.88	0.887	0.050	0.791	1.000
Sab-CD8^+^ T_CM_ cells	108.6	0.928	0.095	0.619	1.000
Sab-CD8^+^T_EM_ cells	400.8	0.487	0.324	0.081	1.000
Sab-CD8^+^ T_EMRA_ cells	1393	0.003	0.427	0.019	0.361
Sab-Intermediate monocytes	503.6	0.119	0.289	0.122	1.000
Sab-Spike-specific B cells	184,329	0.939	0.177	0.350	1.000
Sab-Plasmablasts	-219.2	0.295	-0.106	0.577	1.000

Abbreviations Sab, anti-spike protein antibody; mDC, myeloid dendritic cell; pDC, plasmacytoid dendritic cell; NK, natural killer; CD, clusters of differentiation; T_FH_, follicular helper T; CM, central memory; EM, effector memory; T_EMRA_, terminally differentiated effector memory T cell re-expressing CD45RA

Taken together, the age-related decline in Sab titers is hypothesized to be associated with a decrease in B-cell-lineage cells and other immune cell types, *P* values and slopes are summarized in Table 3. In particular, there is a significant Sab-dependent decrease in the proportion of naive CD4^+^ T cells (*P* = 0.038) and an increase in CD8^+^ T_EMRA_ cells (*P* = 0.003), as shown in Additional file 13. While IGRA responses were maintained or even slightly increased with age, this phenomenon may be attributed to the compensatory maintenance of memory T cell populations, despite the age-related decline in the proportion of naive CD4^+^ and CD8^+^ T cells. The increase in Sab titers after BI may be linked to interactions with these memory T cell populations and T_FH_ cells, warranting further investigation.

## Discussion

The COVID-19 pandemic, a respiratory virus pandemic, emerged a century after the 1918 Spanish flu pandemic, highlighting significant advancements in global health response capabilities [26, 27]. Unlike the Spanish flu, where no vaccines were available, the COVID-19 pandemic saw the rapid development and deployment of multiple vaccines within just one year. This not only curtailed the spread of the virus but also opened avenues for extensive research into vaccine-induced immunogenicity. Unlike other vaccines, which are recommended for specific age groups depending on the risk, primary COVID-19 vaccines are recommended for adults of all ages at the same time. This provided an opportunity to explore the age-dependent immunogenicity of the vaccine in young and middle-aged groups.

Notably, a significant age-dependent decline in Sab titers was observed after vaccination, which became more pronounced with the booster vaccination. Further immune cell profiling revealed a concurrent decline in B cell lineages with age, correlated with a decrease in Sab titers. This suggests that the primary humoral immune response to vaccines wanes from middle age, a finding that has been underemphasized previously. However, after BIs with the Omicron variant, there was a resurgence in the immune response in middle-aged individuals, as evidenced by elevated Sab and PRNT titers. This phenomenon was not solely explained by the analysis of B cell lineages, as the proportion of memory B cells also exhibited a declining trend with age. Instead, preserved memory CD4^+^ T cells and T_FH_ cells, which share epitopes with preexisting common coronaviruses, may explain this phenomenon [28, 29]. In particular, T_FH_ cells, which are pivotal in aiding germinal center formation and the subsequent development of high-affinity antibodies, would be critical to the humoral response after BIs [30]. This reversal of the waning humoral immune response through hybrid immunity explains the decreasing COVID-19 case fatality in the older adult population after the transition to endemicity [31]. Further research should focus on elucidating the signaling pathways and cellular interactions that enable memory T cells to enhance hybrid immune responses, especially in the aged population.

In contrast to the typical decline in immune function with age, the quantitative IFN-γ response did not noticeably deteriorate; instead, it exhibited a weak positive correlation with age. Immune cell profiling provided a reliable explanation, as the proportion of naive T cell subsets decreased with age, whereas the proportion of memory T cell subsets concurrently increased. This shift suggests that differentiated memory T cells may play a compensatory role in sustaining the cellular immune response despite advancing age. However, unlike the humoral immune response, the IFN-γ response in the aged population did not show a greater increase than that of the young population after BIs, suggesting that this compensation of the aging process is limited in proliferative quality [32]. These insights highlight a complex but coordinated adaptation of the immune system with age, characterized by a strategic shift towards memory-based immunity starting in middle age. Although the cohort consisted of healthy individuals, and none of them experienced severe illness after BIs, these age-dependent changes emphasized the need for special consideration of vaccination strategies for middle-aged individuals, particularly those with underlying diseases that might further compromise the immune response.

Incorporating insights from the studies by Gil-Manso et al. [33, 34]. on HCWs vaccinated with mRNA-1273 and BNT162b2 vaccines, and their longitudinal analysis of cellular and humoral responses, our findings are consistent with the observed trends in antibody levels and immune cell profiles. These studies highlighted that older individuals tend to have different kinetics in their immune response compared to younger individuals, especially with mRNA-1273 vaccines showing higher initial antibody levels. This underscores the need for tailored vaccination strategies that consider age-related differences in immune responses.

Our study, while providing valuable insights into age-dependent immunogenicity among healthy individuals, has several limitations. First, the cohort did not include individuals aged 65 years or older, as it consisted of HCWs who had retired before that age. Nevertheless, we observed notable declines in humoral immune responses, even among middle-aged individuals, along with preserved memory T cell function and enhanced hybrid immunity following BIs, which may have mitigated some aging effects. This suggests a broader spectrum of effects of aging on immunogenicity, in addition to the findings in older populations [32]. Second, the present study was conducted as part of a nationwide multicenter cohort, and not all participants underwent comprehensive immunogenicity testing using IGRA, PRNT, and CyTOF. However, the consistency across different immune assessments lends credibility to our findings. Third, our exclusion criteria relied on PCR testing and Nab positivity to identify individuals with prior SARS-CoV-2 infection before mRNA booster administration. This misclassification could potentially impact our findings by underestimating the true incidence of breakthrough infections and their effect on immune responses. To mitigate this issue, future studies should consider regular PCR testing and serological surveys to better identify asymptomatic infections. Additionally, the potential inflation of Type I errors is due to the multiple statistical tests performed. To address this, we applied the Bonferroni correction to our analyses. However, the Bonferroni correction is conservative and may increase the risk of Type II errors, potentially masking true effects. This highlights the need for larger sample sizes in future studies to confirm these findings and provide more power to detect significant effects. Our analysis primarily focused on age as the independent variable. While this provided a clear and interpretable baseline relationship, it is essential to consider other potential confounding variables, such as gender, in future studies to provide a more comprehensive understanding of the observed effects. Finally, immune cell profiling was limited to the pre- and post-booster shot periods, without targeting spike-protein-specific cells, which may have restricted the depth of cellular response interpretation. Future studies should consider a more targeted approach for cell profiling to better understand the specific cellular mechanisms activated by vaccination and infection.

## Conclusions

Our nationwide multicenter longitudinal study of young and middle-aged HCWs elucidated the key aspects of the age-dependent immune response to COVID-19 vaccination and BIs. We observed a decline in vaccine-induced humoral immunity with age, whereas cellular immunity was preserved in the same demographic group. Importantly, our findings suggest that BIs contribute to the development of hybrid immunity, particularly by enhancing the humoral response in middle-aged individuals. This enhanced response is associated with an increase in the proportion of memory T-cell subsets, indicating their crucial role in maintaining immune protection against aging. These insights emphasize the need for tailored vaccination strategies to enhance immune responses in middle-aged populations, especially those with underlying diseases that could compromise their immune responses.

### Electronic supplementary material

Below is the link to the electronic supplementary material.

Supplementary Material 1

## Data Availability

No datasets were generated or analysed during the current study.

## Abbreviations

BAU: Binding antibody units
BIs: Breakthrough infections
BMI: Body mass index
CAS: Cell acquisition solution
COVID-19: Coronavirus disease 2019
CyTOF: Cytometry by time of flight
ELISA: Enzyme-linked immunosorbent assay
HbsAb: Hepatitis B surface antibody
HbsAg: Hepatitis B surface antigen
HCWs: Healthcare workers
IFN-γ: Interferon-gamma
IGRAs: Interferon-gamma releasing assays
IRB: Institutional Review Board
KCDA: Korea Disease Control and Prevention Agency
Nab: Anti-nucleocapsid antibody
PBMCs: Peripheral blood mononuclear cells
PCR: Polymerase chain reaction
PRNTs: Plaque reduction neutralization tests
Sab: Anti-spike protein antibody
SARS-CoV-2: Severe acute respiratory syndrome coronavirus 2
T_CM_: Central memory T cells
T_EM_: Effector memory T cells
T_EMRA_: Terminally differentiated effector memory T cells re-expressing CD45RA
T_FH_: Follicular helper T cells
