# Supplementary material for: Overcoming the age-dependent SARS-CoV-2 vaccine response through hybrid immunity: analysis of humoral and cellular immunity with mass cytometry profiling
Source: Immun Ageing. 2024 Jul 30;21:51. doi: 10.1186/s12979-024-00454-z (PMC11289962; doi:10.1186/s12979-024-00454-z)
Supplement: Supplementary file 1 — Supplementary Material 1 [file 12979_2024_454_MOESM1_ESM.docx]

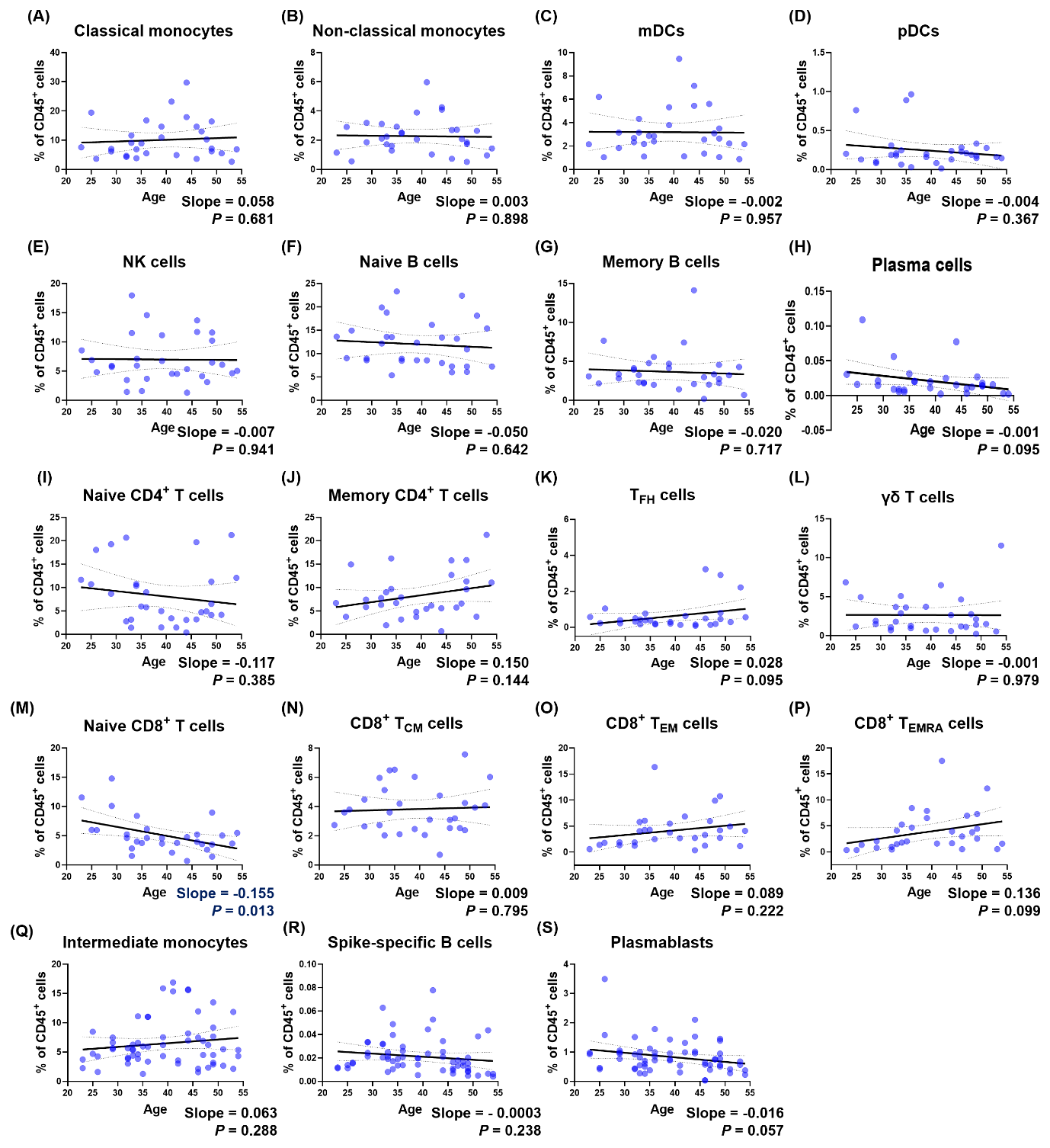

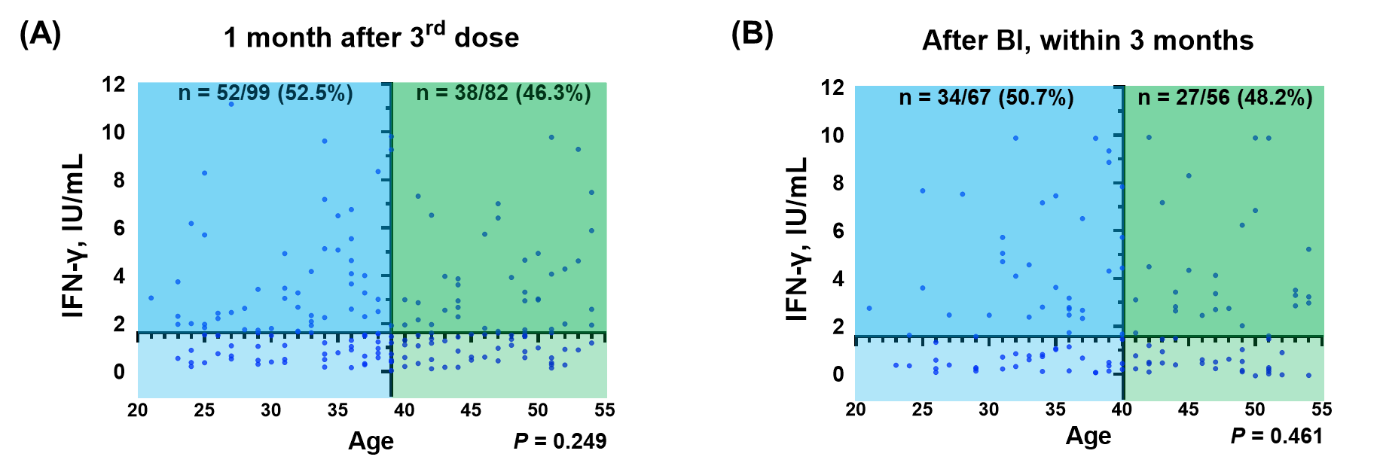
 **File name:** Additional file 1

**File format:** TIFF

**Title of data:** Figure S1.Manual gating strategy for CyTOF analysis.

**Description of data:** This file contains a manual gating strategy.

**File name:** Additional file 2

**File format:** TIFF

**Title of data:** Figure S2. Normality test with Q-Q plot of 347 cohort.

**Description of data:** This file contains Q-Q plots that provide a normality test of whole cohort data.


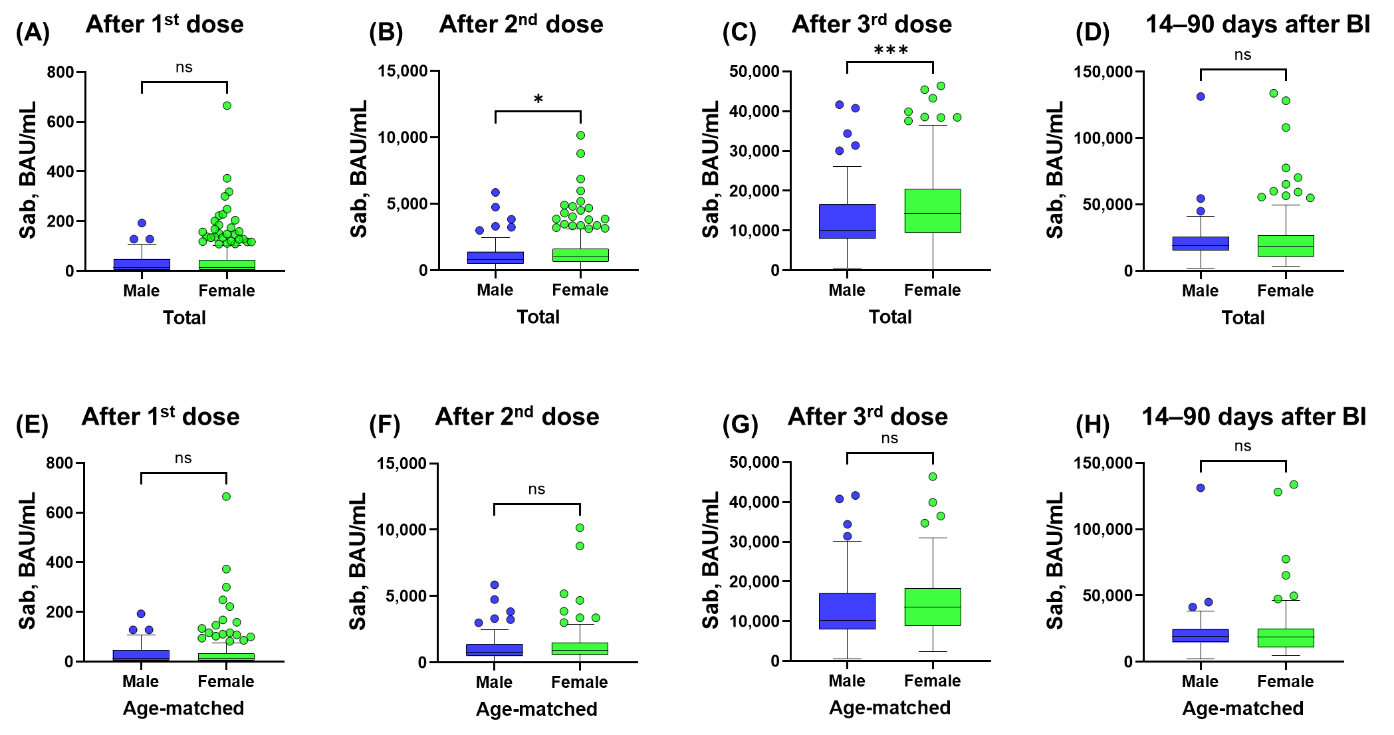
 **File name:** Additional file 3

**File format:** TIFF

**Title of data:** Figure S3. Global view of Sab and IFN- γ levels at different time points

**Description of data:** This file contains violin plots that provide a global view of anti-spike protein antibody (Sab) levels and interferon-gamma (IFN-γ) levels across various time points, independent of age.


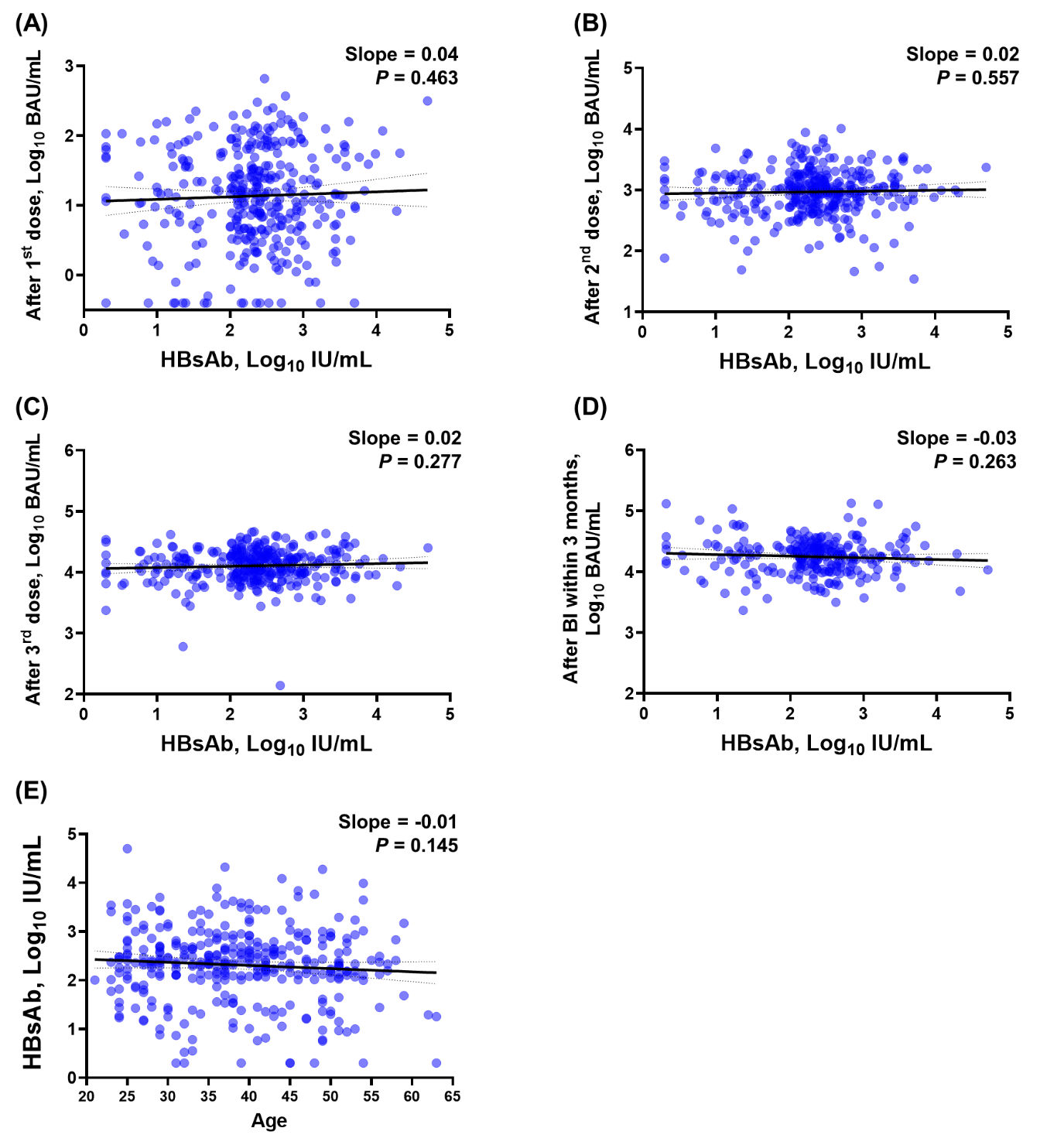
 **File name:** Additional file 4

**File format:** TIFF

**Title of data:** Figure S4. Age-dependent Sab response after COVID-19 vaccinations and BI, waning points.

**Description of data:** This file contains a figure illustrating the age-dependent changes in anti-spike protein antibody levels following COVID-19 vaccinations and breakthrough infections, focusing on the waning points.


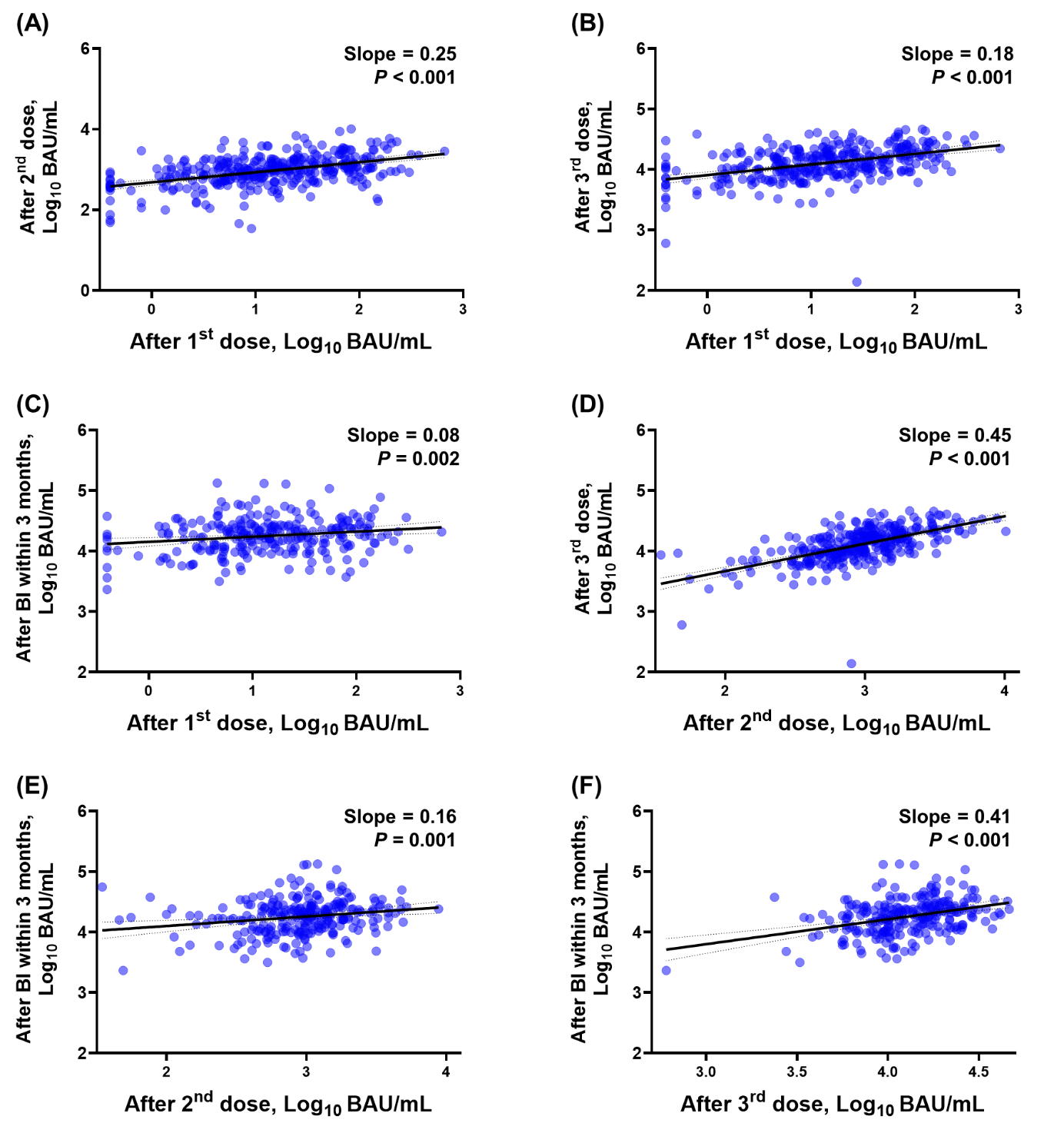

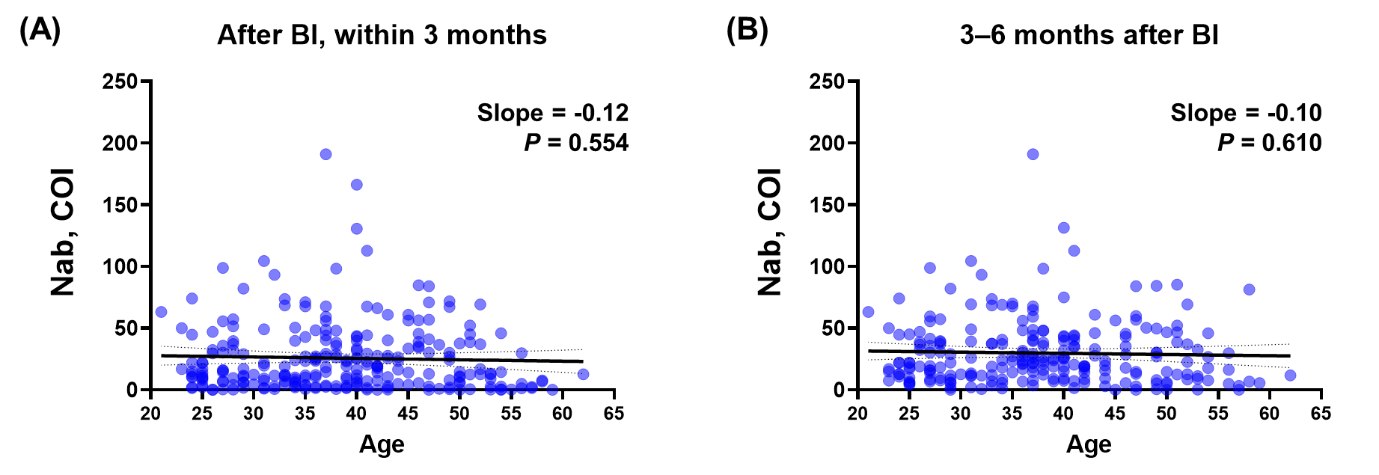
 **File name:** Additional file 5

**File format:** TIFF

**Title of data:** Figure S5. Age-dependent PRNT response after COVID-19 vaccinations and BI.

**Description of data:** This file contains a figure showing the age-dependent plaque reduction neutralization test (PRNT) responses following COVID-19 vaccinations and breakthrough infections.
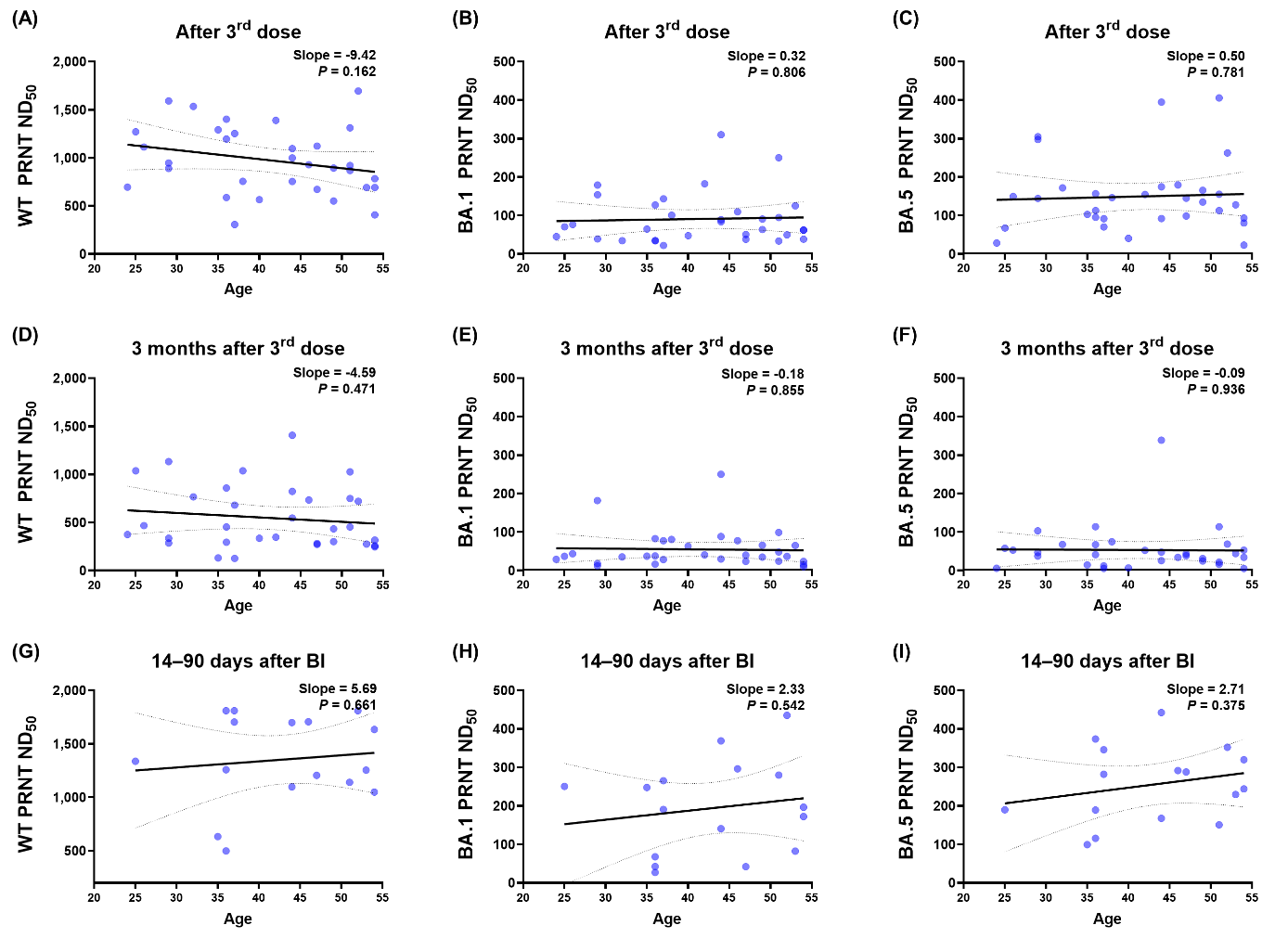
 **File name:** Additional file 6

**File format:** TIFF

**Title of data:** Figure S6. Age-dependent Nab response after a BI.

**Description of data:** This file contains a figure illustrating the age-dependent changes in anti-nucleocapsid antibody (Nab) levels following breakthrough infections.
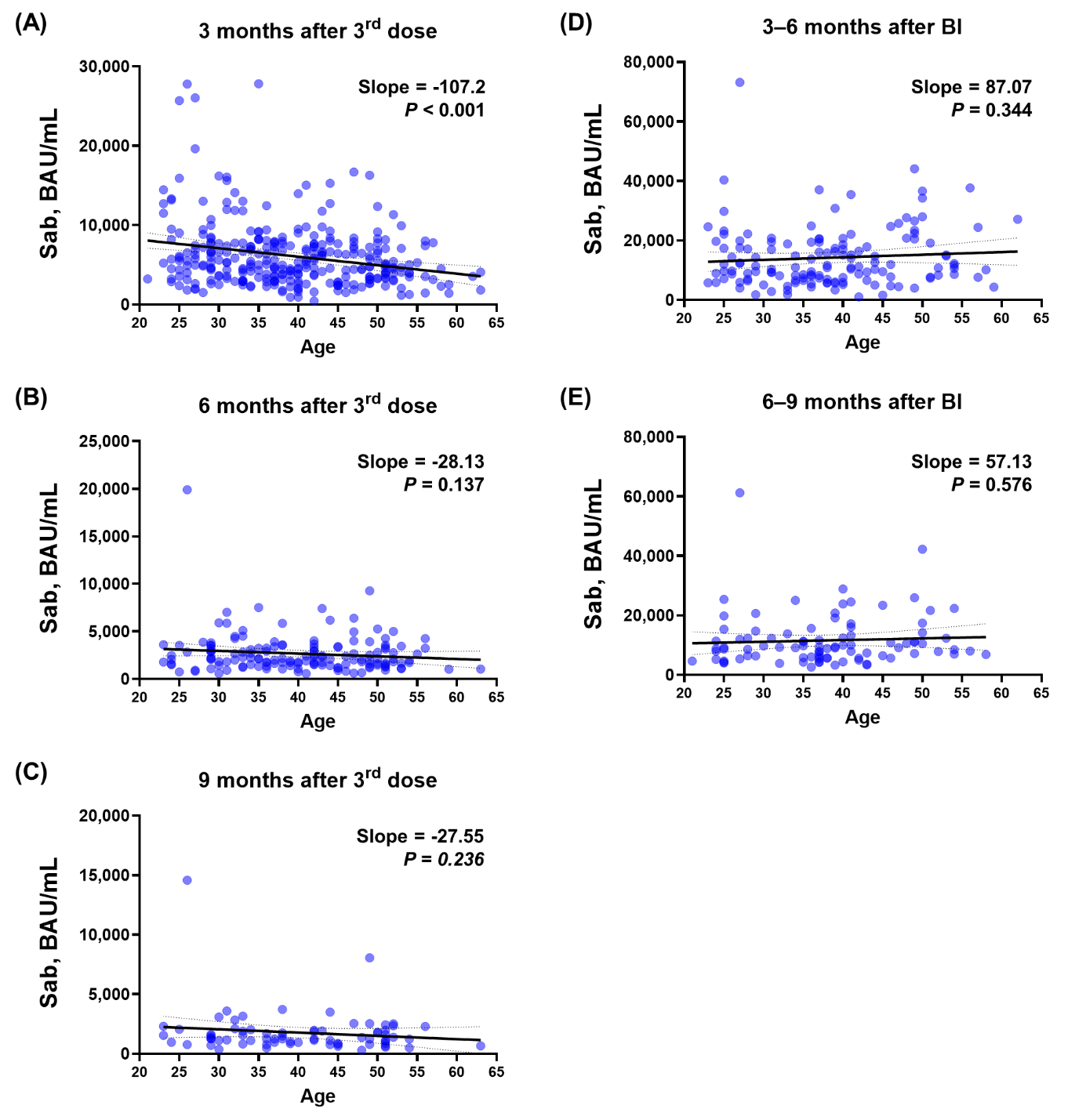
 **File name:** Additional file 7

**File format:** TIFF

**Title of data:** Figure S7. Correlation between Sab levels measured at each antigenic stimulation event.

**Description of data:** This file contains a figure showing the correlation between anti-spike protein antibody (Sab) levels measured at various antigenic stimulation events.

**File name:** Additional file 8

**File format:** TIFF

**Title of data:** Figure S8. Correlation of HBsAb titers with Sab titers and age.

**Description of data:** This file contains a figure illustrating the correlation between hepatitis B surface antibody (HBsAb) titers with anti-spike protein antibody (Sab) titers and age.
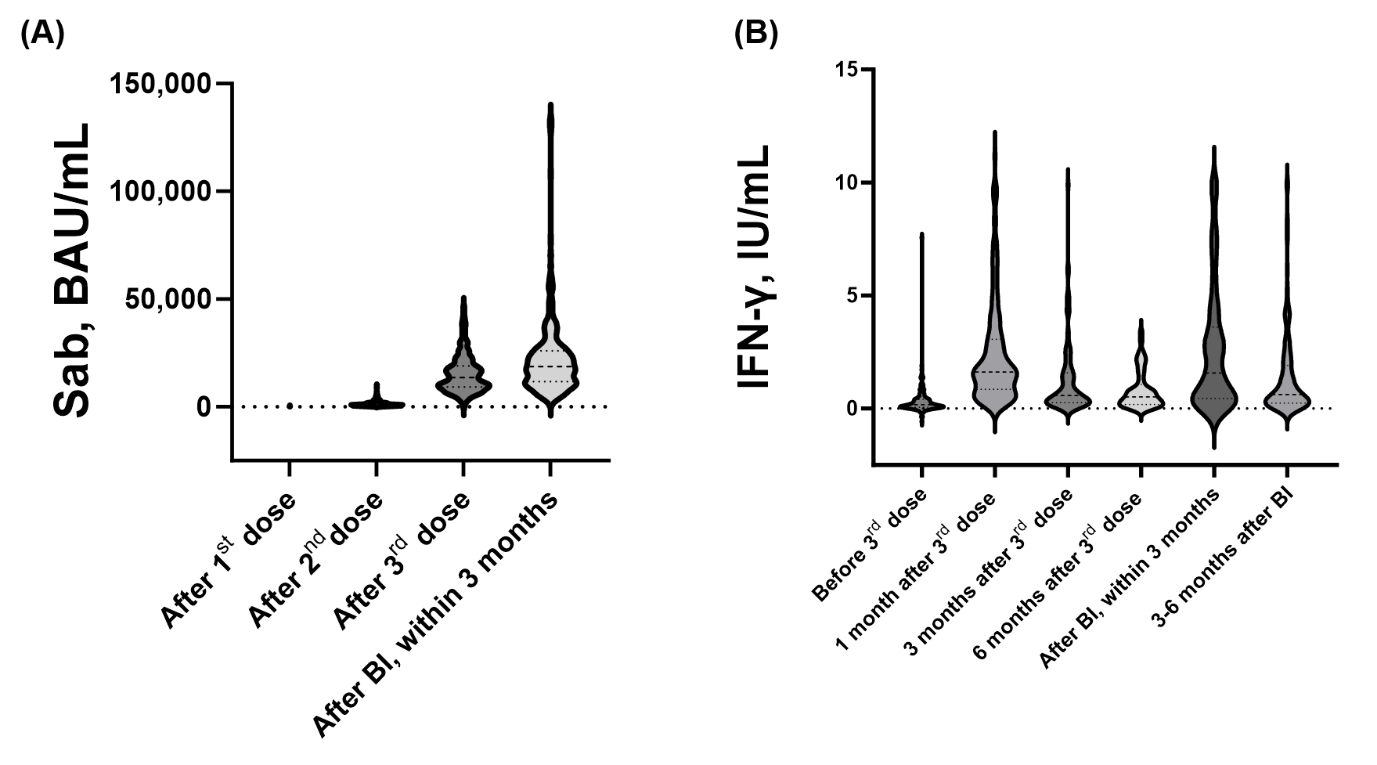

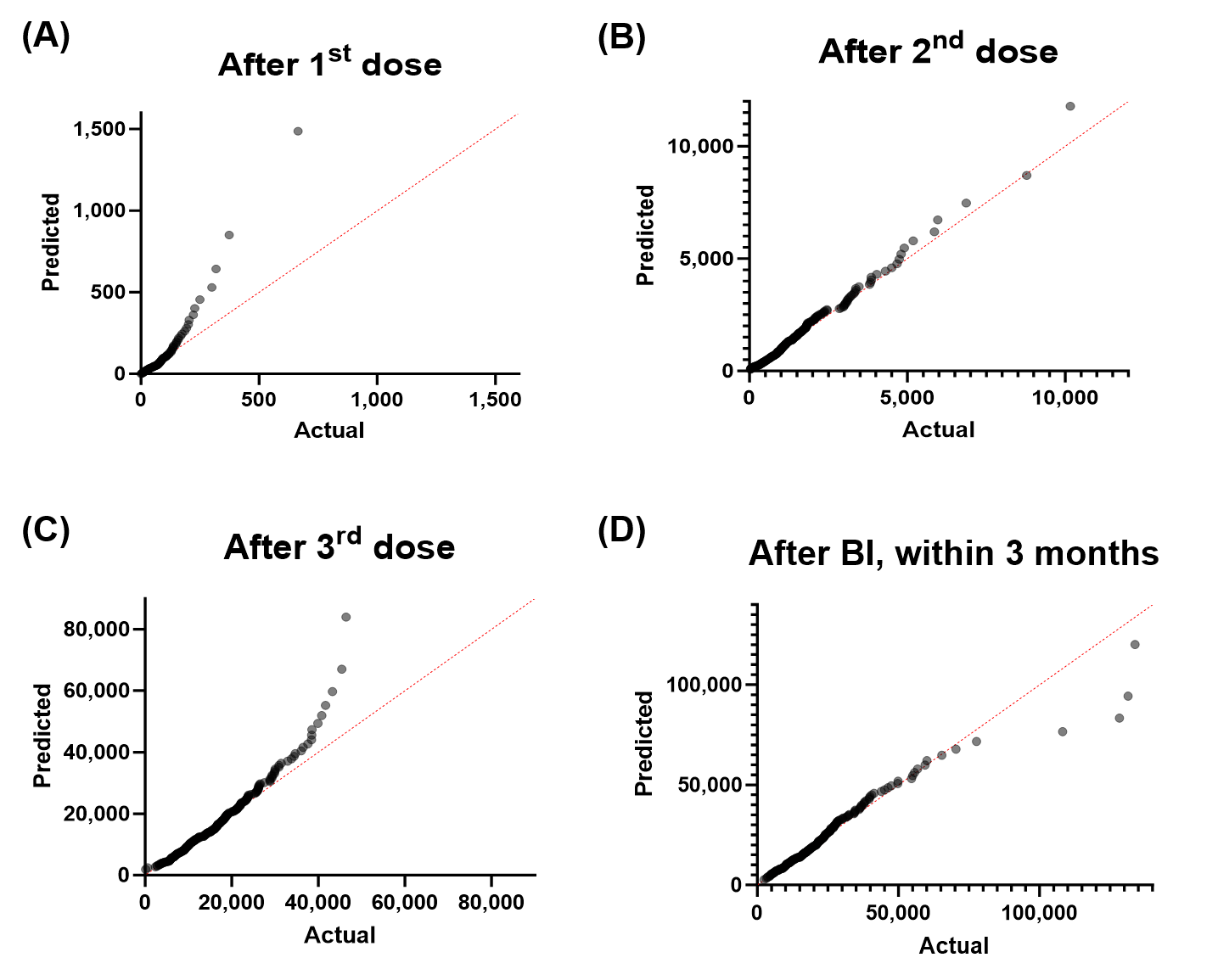
 **File name:** Additional file 9

**File format:** TIFF

**Title of data:** Figure S9. Analysis of potential impact of gender on Sab titers.

**Description of data:** This file contains a figure analyzing the potential impact of gender on anti-spike protein antibody (Sab) titers.


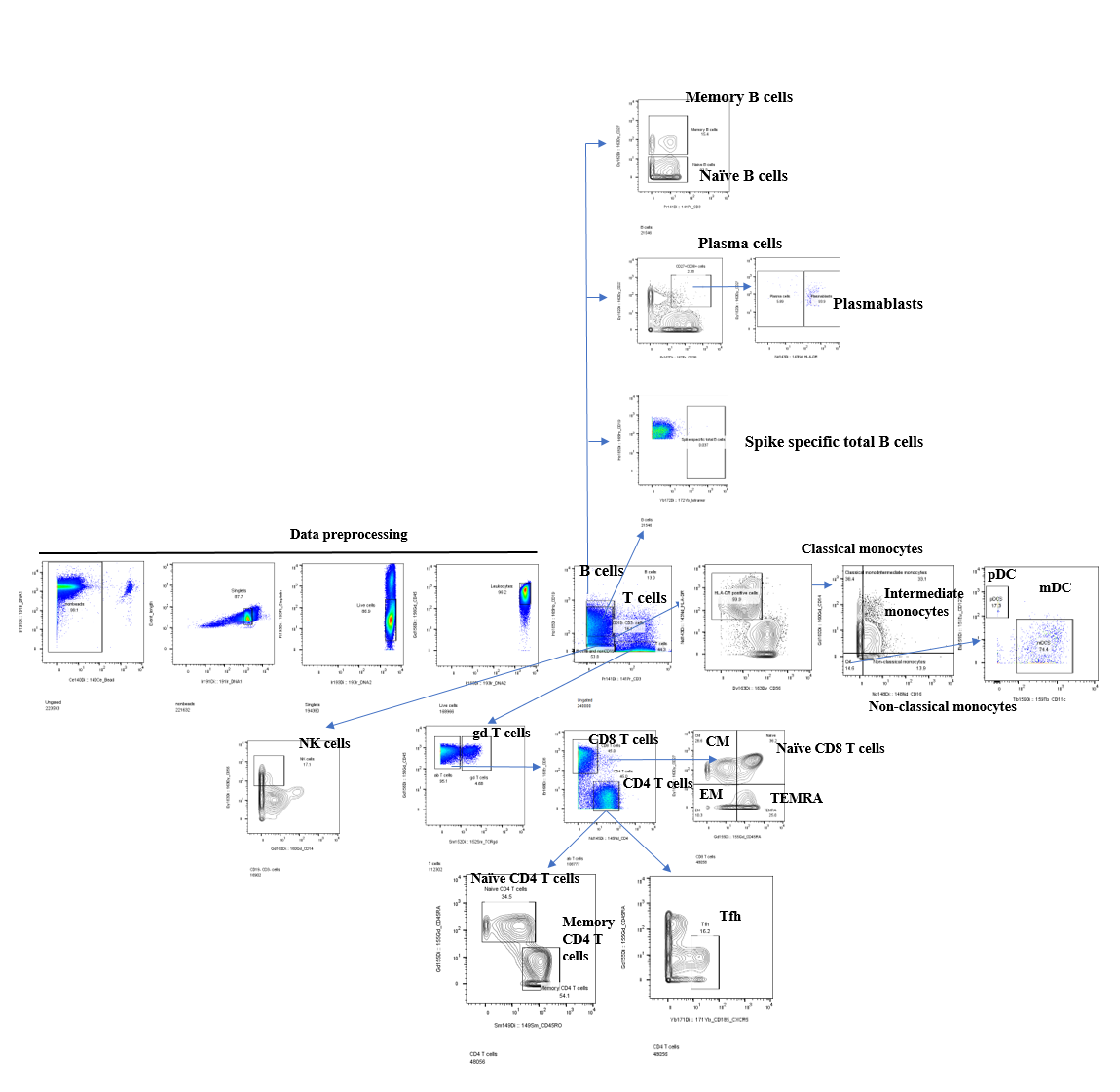
 **File name:** Additional file 10

**File format:** TIFF

**Title of data:** Figure S10. Quadrant charts of IGRA response after the third dose and BI.

**Description of data:** This file contains quadrant charts illustrating the interferon-gamma release assay (IGRA) responses after the third dose of COVID-19 vaccination and following breakthrough infections.

**File name:** Additional file 11

**File format:** TIFF

**Title of data:** Figure S11. Immune cell profiling of immune cells according to age, using multi-parametric CyTOF analysis. (A) classical monocytes, (B) non-classical monocytes, (C) mDCs, (D) pDCs, (E) NK cells, (F) naive B cells, (G) memory B cells, (H) plasma cells, (I) naive CD4^+^ T cells, (J) memory CD4^+^ T cells, (K) T_FH_ cells, (L) γδ T cells, (M) naive CD8^+^ T cells, (N) CD8^+^ T_CM_ cells, (O) CD8^+^ T_EM_ cells, (P) CD8^+^ T_EMRA_ cells, (Q) Intermediate monocytes, (R) Spike-specific B cells, and (S) Plasmablasts.

**Description of data:** This file contains a figure showing immune cell profiling according to age, using multi-parametric CyTOF analysis, focusing on various other cell types.

**File name:** Additional file 12

**File format:** TIFF

**Title of data:** Figure S12. UMAP data visualization of before and after the third dose of vaccination.

**Description of data:** This file contains a figure comparing immune cell profiles before and after the third dose of COVID-19 vaccination at the different age groups.


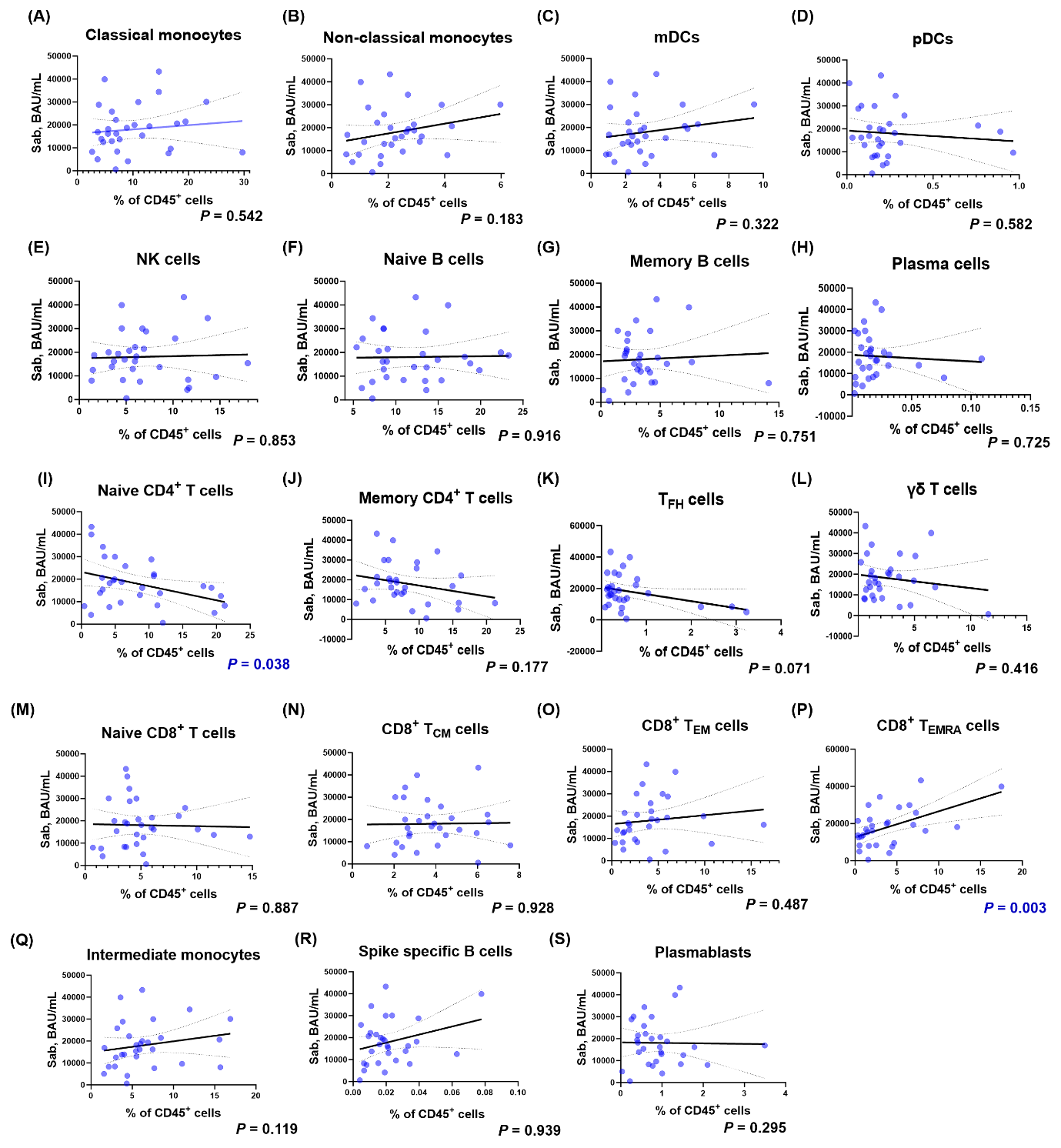
 **File name:** Additional file 13

**File format:** TIFF

**Title of data:** Figure S13. Immune cell profiling according to Sab titer, using multi-parametric CyTOF analysis, before the third dose of vaccination

**Description of data:** This file contains a figure correlation between immune cell subtypes and Sab titer before the third dose of vaccination. (A) classical monocytes, (B) non-classical monocytes, (C) mDCs, (D) pDCs, (E) NK cells, (F) naive B cells, (G) memory B cells, (H) plasma cells, (I) naive CD4^+^ T cells, (J) memory CD4^+^ T cells, (K) T_FH_ cells, (L) γδ T cells, (M) naive CD8^+^ T cells, (N) CD8^+^ T_CM_ cells, (O) CD8^+^ T_EM_ cells, and (P) CD8^+^ T_EMRA_ cells, (Q) Intermediate monocytes, (R) Spike-specific B cells, and (S) Plasmablasts.

Table S1. Marker list for CyTOF analysis

| **Target** | **Metal** | **Target** | **Metal** |
| --- | --- | --- | --- |
| CD3 | 141Pr | CD11c | 159Tb |
| HLA-DR | 143Nd | CD14 | 160Gd |
| CD4 | 145Nd | CD27 | 162Dy |
| IgD | 146Nd | CD56 | 163Dy |
| CD16 | 148Nd | CD161 | 164Dy |
| CD45RO | 149Sm | CD19 | 165Ho |
| CD123 | 151Eu | CD38 | 167Er |
| TCRgd | 152Sm | CD8 | 168Er |
| CD194_CCR4 | 153Eu | CD25 | 169Tm |
| CD45RA | 155Gd | CD185/CXCR5 | 171Yb |
| CD45 | 156Gd | Tetramer | 172Yb |
| IFNg | 158Gd | IL-10 | 166Er |
| TNFa | 175Lu | CXCL10 | 144Nd |
| GranzymeB | 173Yb |  |  |

Table S2. Distribution of samples across different age groups.

| **Age group (years)** | **# of samples** | **Gender (M/F)** | **Average BMI (kg/m^2^)** |
| --- | --- | --- | --- |
| 21–29 | 73 | 9/64 | 21.46 |
| 30–39 | 114 | 29/85 | 22.08 |
| 40–49 | 103 | 19/84 | 22.63 |
| 50–63 | 57 | 25/32 | 23.34 |

Table S3. Statistical analyses using linear regression and spearman correlation of each figure.

| **Figure numbers** | **Correlating variables** | **Linear regression** | |  | **Spearman correlation** | | |
| --- | --- | --- | --- | --- | --- | --- | --- |
|  |  | Slope | *P* value |  | Spearman’s ρ | *P* value | *Bonferroni-corrected*  *P* value |
| **Figure 1** | (A) Age-Sab, after 1st dose | -0.6 | 0.119 |  | -0.082 | 0.129 | 0.517 |
|  | (B) Age-Sab, after 2nd dose | -15.0 | 0.032 |  | -0.130 | 0.016 | 0.062 |
|  | (C) Age-Sab, after 3rd dose | -208.9 | < 0.001 |  | -0.201 | < 0.001 | < 0.001 |
|  | (D) Age-Sab, after BI | 398.8 | 0.001 |  | 0.189 | 0.003 | 0.010 |
| **Figure 2** | (A) Age-IFN-γ, before 3rd dose | 0.01 | 0.030 |  | 0.159 | 0.043 | 0.260 |
|  | (B) Age-IFN-γ, 1mo after 3rd dose | 0.01 | 0.667 |  | 0.005 | 0.946 | 1.000 |
|  | (C) Age-IFN-γ, 3mo after 3rd dose | 0.03 | 0.019 |  | 0.004 | 0.970 | 1.000 |
|  | (D) Age-IFN-γ, 6mo after 3rd dose | 0.00 | 0.814 |  | 0.106 | 0.431 | 1.000 |
|  | (E) Age-IFN-γ, after BI within 3 mo | 0.01 | 0.836 |  | -0.003 | 0.970 | 1.000 |
|  | (F) Age-IFN-γ, 3-6mo after BI | 0.01 | 0.579 |  | -0.043 | 0.614 | 1.000 |
